# Control of lice infestation in horses using a 10 mg/mL deltamethrin topical application

**DOI:** 10.1186/s13620-017-0100-2

**Published:** 2017-06-19

**Authors:** Eloy Castilla-Castaño, Alessandro Vischi, Christelle Navarro, Line Alice Lecru, Claudia Ribeiro, Sophie Pradier, Marie-Christine Cadiergues

**Affiliations:** 1 Dermatology service, Small Animal Hospital, Université de Toulouse, ENVT, 23 chemin des Capelles, 31076 Toulouse, France; 2 Equine Unit, Université de Toulouse, ENVT, 23 chemin des Capelles, 31076 Toulouse, France; 3 Companion Animal Medical Department, Virbac group 13ème rue-LID, 06511 Carros, France; 4 UDEAR, Université de Toulouse, INSERM, ENVT, 23 chemin des Capelles, 31076 Toulouse, France

## Abstract

**Background:**

Two open-controlled studies evaluated the tolerance and the efficacy of a 10 mg/mL deltamethrin-based pour-on solution (Deltanil®; Virbac, France) in treating (study 1) and preventing (study 2) natural *Damalinia equi* infestations in horses. In study 1, seven adult horses received 10 mL of the solution from mane to tail head on day 0 (D0). Four adult horses, living separately, served as non-treated controls. All were naturally infected. Lice burden was recorded by counting the number of live parasites, bilaterally, over seven anatomic regions. Lesional score was based on alopecia, crusts, papules/pustules, nodules/plaques, scales and wounds, each assessed on a 0–3 scale. Evaluation was performed on D0 and subsequently weekly until D56 in treated horses and on D0 and D56 in control horses. In study 2, six adult horses free of parasites were similarly treated on D-2 and D30. Two adult horses, naturally infested with *D. equi* and left untreated, were mixed with the treated horses from D0 to D60. Evaluation was performed similarly to study 1 on all horses, fortnightly until D60.

**Results:**

No adverse event was recorded in either study. In study 1, parasite and lesional scores of control horses were maintained on D56. Parasite scores of the treated horses were reduced by 98% on D7 and 100% from D15 to D56 (mean [SD]: D0 44 [58.4]). Lesional score in treated horses was reduced by 24, 82, 47, 91, 96, 93, 93 and 100% on D7, 15, 21, 28, 35, 42, 50 and 56, respectively (mean [SD]: D0 3.1 [1.8]).

In study 2, the lice populations remained high in the two control horses throughout the study (*max* mean [SD]: D0 159 [151.3], *min* D45 34 [39.6]). On treated animals, all parasite counts were negative except on D15 (one louse found). The protection rate was 99.7% on D15 and 100% from D30 to D60.

**Conclusions:**

A single application of the 10 mg/mL deltamethrin preparation was effective and safe in the treatment and in the prevention of lice infestation in these horses. It was also effective in preventing new infestations for one month.

## Background

Lice infestation, also called phtyriasis or pediculosis, is fairly common worldwide in horses [1]. It is more often a winter or early spring skin disease favored by long hair coat and animal promiscuity. Unbalanced feeding and/or concurrent diseases can also be contributing factors. It usually shows as a pruritic, alopecic and scaling skin disease [2–4]. However, skin lesions are not necessarily related with the parasite burden [1]. Horses can be parasitized by chewing lice (Mallophagan; *Damalinia equi*, synonyms *Bovicola equi*, *Wernekiella equi equi*) and or sucking lice (Anoplura, *Haematopinus asini*) [5]. *D. equi* is mostly encountered on the forehead, neck and dorso-lateral trunck whereas *H. asini* is more commonly isolated from the mane, base of tail, on the fetlocks and upper and inner thighs [2]. However, in some studies this distribution was not as clear [1]. Their presence in the haircoat is a potential source of discomfort. Heavy infestation of sucking lice may cause anemia. Infestation is usually by direct contact between horses; however indirect transfer via a blanket or any piece of equipment may occur. The diagnosis of phtyriasis is easy and based on clinical signs combined with gross evidence of parasites. The literature is rather sparse regarding lice control in horses. The use of imidacloprid [6, 7], phoxim [6], selenium sulfide [8], triflumuron [9], permethrin combined with dimilin [10] or pyriproxyfen [4], fipronil [3] and neem seed extract [11] has been described as effective in controlling equine phtyriasis. We report here the use of a 10 mg/mL deltamethrin-based pour-on solution (Deltanil®; Virbac, Carros, France) in two open clinical field studies. Study 1 aimed to evaluate its efficacy on infested horses; in study 2, the efficacy of the product in preventing new infestations was investigated.

## Methods

### Animals

Adult leisure horses, in good health, not destined for human consumption, naturally infested with *D. equi* (studies 1 and 2) or lice-free (study 2) and living permanently in outdoor conditions in the southwest of France were included. They were kept permanently outdoors on pasture grass and received hay as a complement. They were provided water *ad libitum* with a self-filling trough. Throughout the studies, management conditions were to be kept identical. No antiparasitic or anti-inflammatory medication was allowed.

As phtyriasis is a seasonal skin disease, horses living separately, but in the same geographical area, were included in parallel and left untreated, serving as sentinels, to ensure that the lice population would not decrease spontaneously throughout the study (study 1). In study 2, naturally infested horses, left untreated, were introduced at day 0 (D0) to the treated group and left within the herd for the duration of the study, to serve as a natural source of lice. Owner consent was obtained prior to beginning the studies.

### Product

As this product is not licensed in horses, the manufacturer’s instructions on dosage to cattle were used and a 10 mg/mL deltamethrin-based pour-on (Deltanil®; Virbac, Carros, France) on the single dose of 10 mL per animal was applied from mane to tail head at D0 (study 1), D-2 and D30 (study 2) after parasite and lesional score evaluations.

### Evaluation methods

Parasite score was recorded according to the European Medicines Agency’s method for ruminants [12] parting the coat bilaterally, over the neck, shoulder, withers, barrel, buttock and quarters and counting the number of live parasites on D0, 7, 14, 21, 28, 35, 42, 50 and 56 in study 1 and D-2, 15, 30, 45 and 60 in study 2.

Lesional score was based on alopecia, crusts, papules/pustules, nodules/plaques, scales and wounds. Each lesion type was scored independently on a 0–3 scale (0 = none; 1 = mild; 2 = moderate; 3 = severe; maximal score = 18) on the same days as parasite enumeration, this was always performed by the same investigators.

### Data analysis

In study 1, the response to treatment was assessed as the change from baseline to each examination day for parasite and lesional scores. Wilcoxon signed-rank tests were used to compare data obtained on each examination day with the D0 baseline. In study 2, the percentage of protection was calculated using Abbott formula:

$$ \% of\; protection=\frac{\left( mean\; number\; of\; lice\; in\; non- treated\; horses\right)-\left( mean\; number\; of\; lice\; in\; treated\; horses\right)}{mean\; number\; of\; lice\; in\; non- treated\; horses} $$

A bilateral Mann–Whitney test was used to compare both samples.

Significance was defined as *P*<0.05. All statistical analysis was performed using XLSTAT 2017–02 (Addinsoft SARL, Paris, France).

## Results

### Study 1

Seven adult horses from the same herd - six mares and one gelding, between seven and 24 years old and weighing between 428 and 567 kg - were included. Four horses – one mare and three geldings, between nine to 25 years old and weighing between 422 and 660 kg - living separately in the same geographic area and held in the same conditions, served as non-treated controls on days 0 and 56.

**Fig. 1:**
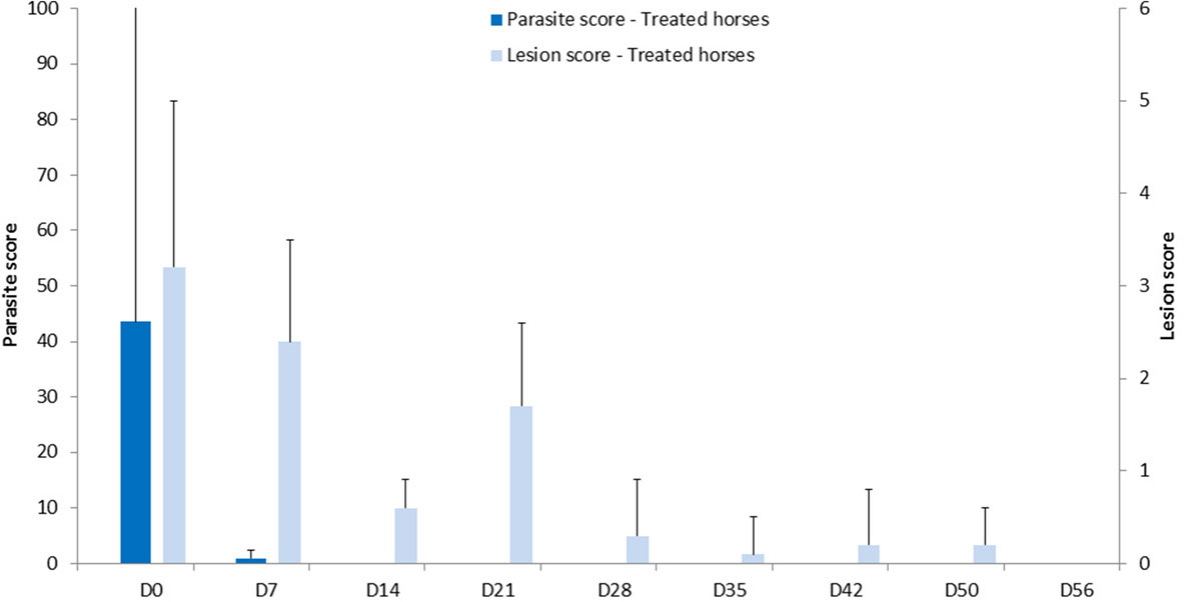
Arithmetic mean and standard deviation of parasite scores and lesional scores in horses treated with a 10 mg/mL deltamethrin topical application in study 1 (efficacy on lice-infested horses). Treatments were applied at D0

In control horses, D56 parasite and lesional scores were comparable to those observed on D0. Arithmetic means [SD] of parasite and lesional scores were 46 [8.5], 3 [0] on D0 and 51 [15.6], 3 [0] on D56; respectively. Parasite scores of treated horses were reduced by 98% on D7 (5 horses were lice-free) and 100% from D14 to D56 (mean [SD]: D0 44 [58.4]). Lesional scores in treated horses were reduced by 24%, 82%, 47%, 91%, 96%, 93%, 93% and 100% on D7, 14, 21, 28, 35, 42, 50 and 56, respectively (mean [SD]: D0 3.1 [1.8]) – Fig. 1. All scores were significantly reduced compared to D0 (*p*<0.05) except lesional score on D7 (*p* = 0.1).

### Study 2

**Fig. 2:**
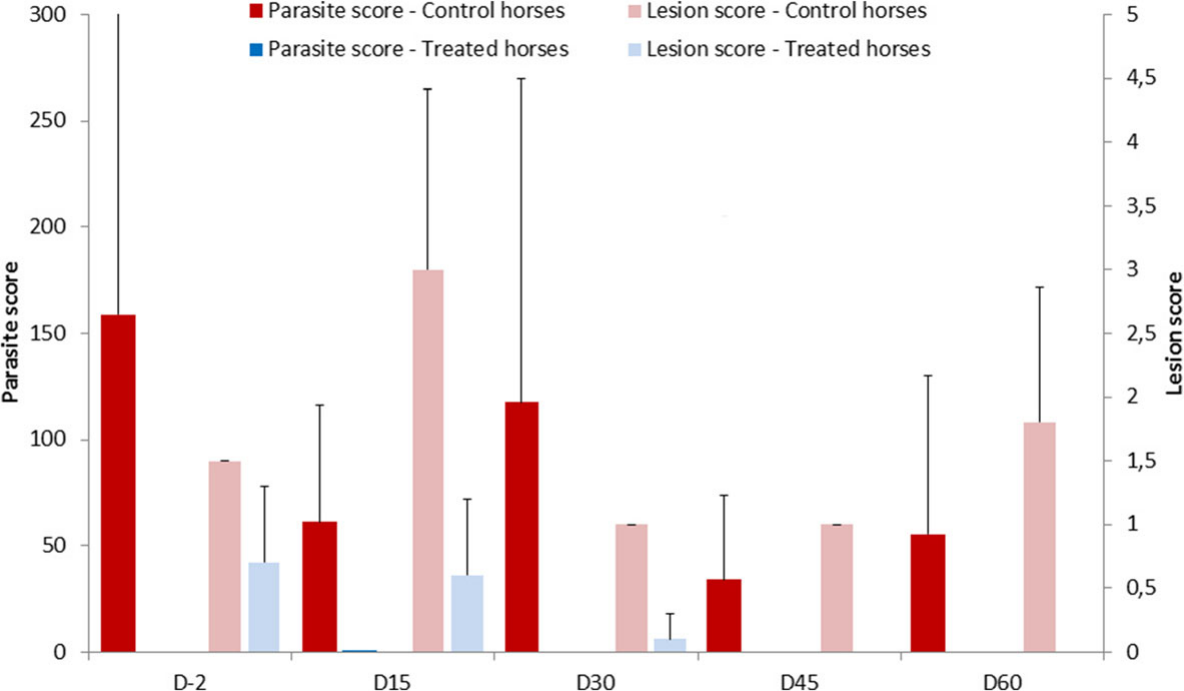
Arithmetic mean and standard deviation of parasite scores and lesional scores in control horses and in horses treated with a 10 mg/mL deltamethrin topical application in study 2 (prevention of lice-infestation in lice-free horses). Treatments were applied at D-2 and D30

Two gelding horses, 21 and 24 years old, weighing 502 and 503 kg and harboring 52 and 266 lice, respectively, were introduced at D0 in a herd composed of six adult horses – five mares and one gelding, between eight and 25 years old and weighing between 436 and 601 kg. On D-2, after a thorough parasitological examination revealing the total absence of lice, the six horses received an individual treatment (*see product section in M&Ms*). Lice population remained high in the two control horses throughout the study, maintaining an acceptable and permanent source of infestation: arithmetic means [SD] were 61 [55.2] on D15, 117.5 [152] on D30, 34 [39.6] on D45 and 55.5 [74.2] on D60 (Fig. 2). Lesional score of the two infested horses remained relatively steady, between 1 (D30 and 45) and 3 (D15). All parasite counts performed on treated animals were negative except on D15 when a single parasite was found. The protection rate was 99.7% on D15 and 100% from D30 to D60. Differences between the two populations were significant (*p*<0.05) throughout the study. Four horses had minor lesions on D-2, their intensity decreased on D15 and D30 and lesions were no longer visible on D45 and D60 (Fig. 2).

## Discussion

Deltamethrin, a type II pyrethroid, has been successfully used to control lice in cattle [13, 14], sheep [15] and goats [16] and is licensed for this purpose in various countries. The deltamethrin formulation which was used in the present report is licensed in Europe for flies and lice in dairy and beef cattle and ticks, lice, keds and blowfly strike in sheep. It can also be used against lice and ticks in lambs. Deltamethrin efficacy has been demonstrated in horses against midges [17], louse flies [18] and tsetse flies [19]. To our knowledge, no published data are available against equine lice.

The 13 horses who were treated tolerated a single application (7 horses) or two applications, a month apart (6 horses), very well. Two previous studies had already shown the excellent tolerance to a daily application of a 10 mL dose for seven consecutive days (McGahie D and Navarro C, unpublished observations) and a weekly application of the same dose for five consecutive weeks (Navarro C, Casamatta J and Viaud S, unpublished observations). A single application exhibited a rapid and prolonged activity in infested animals: after 7 days, only two horses were still positive, and the lice burden was very low (3 lice each) despite a high level of initial infestation and the absence of known ovicidal efficacy.

In study 1, we elected to include a control group to ensure that the reduction of the lice population in horses receiving the treatment was not spontaneous. In the published studies investigating an insecticidal activity against lice, several had no control group [3, 4, 6, 11]. As phtyriasis is mainly a seasonal parasitic disease, in the absence of a control group, the absence of parasites on treated animals could be misinterpreted. Parasite scores of the control horses were maintained throughout the study allowing interpretation of the results in the treated group. In study 2, the control horses were observed throughout the study being in close contact with the treated horses, and therefore a possible direct source of infestation. Conversely, insecticidal product could possibly transfer by contact from treated horses to control animals. This could explain the relative decrease of lice numbers, particularly on one animal. Nevertheless, this was not sufficient to exterminate the entire population on the non-treated horses. Therefore, it is recommended to treat the entire group when lice infestation is identified, even if dermatological examination appears unremarkable, since as previously reported [1], and confirmed in these studies, skin lesions are not necessarily related with the parasite burden. In study 2, four treated horses had minor lesions, with a score comprised between 0.5 and 1.5 whilst the maximum possible score was 18. Lesions were non-specific and since horses were free of lice, lesions could be due to unforeseen reasons such as environmental traumas, biting flies or even fights between horses. The fact that their intensity decreased and became nil supports this hypothesis. In control horses of study 2, changes of lesional scores could be explained by changes in pruritus intensity, as most of the lesions were self-inflected lesions, such as alopecia. As it is commonly observed when treating a parasitic skin disease, independently of the animal species or the parasite, parasitological cure preceeds clinical cure. In study 1, skin lesions decreased progressively, but slower than the parasite score. It took one month before obtaining a >90% reduction of skin lesions in treated animals.

It is commonly accepted that insecticidal products have a shorter duration of efficacy in horses compared to livestock. A likely explanation is the abundant sweating in equine species. The horses who were included in those studies were kept outdoors but did not race or exercise. Furthermore, lice are permanent parasites, which implies a permanent contact with hair and skin and therefore a much more sustainable efficacy of insecticides compared with flying insects, e.g. midges. This means that for other parasites and/or in exercising/racing horses, such a product might have to be applied more frequently than once monthly, but this would need to be confirmed with appropriate studies.

In conclusion, in these horses, in field conditions, the 10 mg/mL deltamethrin-based pour-on solution appeared to be effective, safe and practical in the treatment of lice infestation. It was also effective in preventing new infestations for one month.

## Abbreviations

D0: Day 0
Dx: Day x
SD: standard deviation

## References

[1] Larsen KS, Eydal M, Mencke N, Sigurdsson H (2005). Infestation of *Werneckiella equi* on Icelandic horses, characteristics of predilection sites and lice dermatitis. Parasitol Res.

[2] Bergvall K (2005). Advances in acquisition, identification, and treatment of equine ectoparasites. Clin Tech Equine Pract.

[3] Da Silva A, Tonin A, Lopes L (2013). Outbreak of lice in horses: epidemiology, diagnosis, and treatment. J Equine Vet Sci.

[4] Sorrell MS, Fish RE, Taylor KH (2010). Pediculosis in two research ponies (*Equus caballus*). J Am Assoc Lab Anim Sci.

[5] Wright R (1999). Lice on horses. Can Vet J.

[6] Mencke N, Larsen KS, Eydal M, Sigurdsson H (2004). Natural infestation of the chewing lice *(Werneckiella equi)* on horses and treatment with imidacloprid and phoxim. Parasitol Res.

[7] Mencke N, Larsen KS, Eydal M, Sigurethsson H (2005). Dermatological and parasitological evaluation of infestations with chewing lice (*Werneckiella equ*i) on horses and treatment using imidacloprid. Parasitol Res.

[8] Paterson S, Orrell S (1995). Treatment of biting lice (*Damalinia equi*) in 25 horses using 1% selenium sulfide. Equine Vet Educ.

[9] Lowden S, Gray S, Dawson K (2007). Treatment of natural infestations of the biting louse (*Werneckiella equi*) on horses using triflumuron, a benzoylurea derivative insect growth regulator. Vet Parasitol.

[10] Reeves WK, Miller MM (2009). Control of *Bovicola equi* (Phthiraptera: Trichodectidae) with Dimilin and permethrin. J Vector Ecol.

[11] Al-Quraishy S, Abdel-Ghaffar F, Al-Rasheid KA, Mehlhorn J, Mehlhorn H (2012). Observations on effects of a neem seed extract (MiteStop(R)) on biting lice (mallophages) and bloodsucking insects parasitizing horses. Parasitol Res.

[12] EMEA/CVMP/625/03 final. http://www.ema.europa.eu/docs/en_GB/document_library/Scientific_guideline/2009/10/WC500004643.pdf. Assessed 30 May 2017.

[13] Rothwell JT, Hacket KC, Ridley I, Mitchell L, Donaldson C, Lowe LB (1999). Therapeutic efficacy of zeta-cypermethrin pour-on for the treatment of biting and sucking lice in cattle under field conditions. Aust Vet J.

[14] Titchener RN (1985). The control of lice on domestic livestock. Vet Parasitol.

[15] Morcombe PW, Gardner JJ, Millar LE, Wilkinson FC, De Chaneet GC, Devereaux DJ (1992). The efficacy of synthetic pyrethroid insecticides applied to the backline of sheep against four strains of lice (*Damalinia ovis*). Aust Vet J.

[16] Brown L, van der Linde TC, Fourie LJ, Horak IG (2005). Seasonal occurrence and production effects of the biting louse *Damalinia limbata* on Angora goats and 2 treatment options. J S Afr Vet Assoc.

[17] Robin M, Archer D, McGowan C, Garros C, Gardes L, Baylis M (2015). Repellent effect of topical deltamethrin on blood feeding by *Culicoides* on horses. Vet Rec.

[18] Parashar BD, Gupta GP, Rao KM (1991). Control of the haematophagous fly *Hippobosca maculata*, a series pest of equines, by deltamethrin. Med Vet Entomol.

[19] Gouteux JP, Le Gall F, Guillerme JM, Demba D (1996). Insecticide treatment (Pour on and Spot on) of cattle against *Glossina fuscipes fuscipes* in the Central African Republic. Vet Res.

